# *Mycoplasma genitalium* Lipoproteins Induce Human Monocytic Cell Expression of Proinflammatory Cytokines and Apoptosis by Activating Nuclear Factor *κ*B

**DOI:** 10.1155/2008/195427

**Published:** 2008-04-23

**Authors:** Yimou Wu, Hong Qiu, Yanhua Zeng, Xiaoxing You, Zhongliang Deng, Minjun Yu, Cuiming Zhu

**Affiliations:** Pathogenic Biology Institute, University of South China, 421001 Hengyang, China

## Abstract

This study was designed to investigate the molecular mechanisms responsible for the induction of proinflammatory cytokines gene expression and apoptosis in human monocytic cell line THP-1 stimulated by lipoproteins (LPs) prepared from *Mycoplasma genitalium*. Cultured cells were stimulated with *M*. *genitalium* LP to analyze the production of proinflammatory cytokines and expression of their mRNA by ELISA and RT-PCR, respectively. Cell apoptosis was also detected by Annexin V-FITC-propidium iodide (PI) staining and acridine orange (AO)-ethidium bromide (EB) staining. The DNA-binding activity of nuclear factor-*κ*B (NF-*κ*B) was assessed by electrophoretic mobility shift assay (EMSA). Results showed that LP stimulated THP-1 cells to produce tumor necrosis factor-*α* (TNF-*α*), interleukin-1*β* (IL-1*β*), and IL-6 in a dose-dependent manner. The mRNA levels were also upregulated in response to LP stimulation. LPs were also found to increase the DNA-binding activity of NF-*κ*B, a possible mechanism for the induction of cytokine mRNA expression and the cell apoptosis. These effects were abrogated by PDTC, an inhibitor of NF-*κ*B. Our results indicate that *M*. *genitalium*-derived LP may be an important etiological factor of certain diseases due to the ability of LP to produce proinflammatory cytokines and induction of apoptosis, which is probably mediated through the activation of NF-*κ*B.

## 1. INTRODUCTION

*Mycoplasma genitalium* is a cell wall-less bacterial pathogen with the distinction of possessing the smallest known genome of any self-replicating cells, it was discovered in 1981 when isolated from the urethra of two men with nongonococcal urethritis [1]. The exact mode of infection and pattern of diseases caused by *M. genitalium* still remains to be solved, but the pathogen is presumed to be sexually transmitted, and the infections often appear to be chronic and asymptomatic [2, 3]. Most investigations have concerned male urethritis patients but *M. genitalium* has also been implicated in pelvic inflammatory disease, pneumonia, arthritides, and AIDS [4–6].

It is known that *M. genitalium* adheres to host cells by the terminal tip organelles, which is the first step for the pathogenicity. However, the exact molecular pathogenesis of *M. genitalium* is vague [7]. It has been suggested that during mycoplasmal infection, the damage to host cells is not caused by direct lesion but by immunopathogenesis [8–10]. Lipoproteins (LPs) are important proteins exposed on the surface of *M. genitalium*. With the intimate interaction with the host cells, LP could influence the functions of monocytes, macrophages, and brain astrocytes, then lead to proinflammatory cytokine production or, in particular cases, to necrosis or apoptosis [11]. For example, *M. fermentans* lipid extract of protein kinase K-digested LP can induce macrophages to release proinflammatory cytokines such as tumor necrosis factor-*α* (TNF-*α*), interleukin-1*β* (IL-1*β*), and IL-6 by activating nuclear factor-*κ*B (NF-*κ*B) and activator protein 1 [12].

Recently, it has been found that mycoplasmal lipoproteins and lipopeptides are capable for leading to apoptoic cell death in macrophage through toll-like receptor 2 (TLR2) and TLR6 [13, 14]. Signaling by TLR2 leads to an activation of nuclear transcription factor NF-*κ*B and an induction of NF-*κ*B-controlled genes such as Bcl-Xs, Bax, and Bad after its activation [15]. NF-*κ*B activation may have a two-fold influence on cell apoptosis, either inhibiting apoptosis or accelerating cell death, which depends on different cell lines and different stimuli.

No research has been reported on the pathogenicity of *M. genitalium* to human monocytes, however; and we have studied whether *M. genitalium* LP could induce human monocytes cell line THP-1 to express proinflammatory cytokines and apoptosis. Therefore, the present study is aimed to investigate whether *M. genitalium* LP-induced apoptosis in monocytes is associated with the activation of NF-*κ*B, and evaluate the effect of the specific inhibitor pyrrolidine ditheiocarbamate (PDTC) on the production of proinflammatory cytokines and apoptosis.

## 2. MATERIALS AND METHODS

### 2.1. Cell culture and stimulation

The human monocytic cell line THP-1 (China Center for Type Culture Collection, Wuhan University, Wuhan, China) was cultured in RPMI 1640 medium (Hyclone, Utah, USA) supplemented with 2 mmol/L L-glutamine and 10% heat-inactivated fetal bovine serum (FBS, Hyclone, USA) at 37°C with 5% CO_2_. Cells were cultivated in 1% FBS overnight in 24-microwell plates (Costor, NY, USA) at a concentration of 10^5^ cells/mL before stimulation. 0.5 to 5 *μ*g/mL of LP or 0.1 *μ*g/mL of lipopolysaccharide (LPS, Sigma-Aldrich, Mo, USA) was added into the medium for 24 hours. To evaluate the effect of PDTC, cells were preincubated with 25 *μ*M PDTC (Sigma-Aldrich) for 30 minutes before stimulation.

### 2.2. Mycoplasma culture and LP preparation

*M. genitalium* (G-37 strain, ATCC) was cultivated in modified SP-4 medium until the beginning of the stationary phase and then pelleted by centrifugation. The preparation of LP and the aqueous phase (used as a control) followed the methods described previously [16]. Lipoproteins in the Triton X-114 phase were precipitated by methanol and used for stimulation after being suspended in sterile PBS. The protein concentration was determined by using a bicinchoninic acid kit (Pierce, Oud Beijerlands, the Netherlands). The preparations were preincubated with 100 *μ*g/mL polymyxin B for 2 hours prior to stimulation in order to eliminate the possibility of endotoxin contamination during the process of its preparation.

### 2.3. Assay for cytokine detection

THP-1 cells were cultivated and stimulated in 24-well tissue culture plates as described above. Being stimulated after 24 hours, the cells were lysed by two consecutive cycles of freezing/thawing, thus the samples represented the total amount of cytokines produced (both intracellular and those that have released into the supernatant). The cytokines concentration was measured by using human TNF-*α*, IL-1*β*, IL-6 ELISA kits (Jingmei Biotech, Shenzhen, China). Supernatants were used for cytokine determination by the quantitative “sandwich” ELISA technique, using monoclonal antibodies specific for the tested cytokines. The detection limit of the ELISA kits for TNF-*α*, IL-1*β*, and IL-6 were 7 pg/mL, 2 pg/mL, and 2 pg/mL, respectively. The assays were performed in accordance with the manufacturer's instructions.

### 2.4. Detection of cytokine expression by RT-PCR

Total RNA was extracted from the treated THP-1 cells with Trizol reagent (Invitrogen Life Technologies, Calif, USA). First-strand cDNA synthesis from the total RNA was performed by using AMV reverse transcriptase XL (TaKaRa, Dalian, China). Primer sequences and sizes of the PCR products were as follows: *β*-actin (564 bp):5′ -CTG GGA CGA CAT GGA GAA AA-3′ (forward primer) and 5′ -AAG GAA GGC TGG AAG AGT GC-3 (reverse primer); IL-1*β* (252 bp): 5′ -TAT TAC AGT GGC AAT GAG G-3′ (forward primer) and 5′ -ATG AAG GGA AAG AAG GTG-3′ (reverse primer); TNF-*α* (324 bp): 5′ -CAG AGG GAA GAG TTC CCC AG-3′ (forward primer) and 5′ -CCT TGG TCT GGT AGG AGA CG-3′ (reverse primer); and IL-6 (430 bp): 5′ -TGA CCC AAC CAC AAA TGC-3′ (forward primer) and 5′ -CGA GCT CTG AAA CAA AGG AT-3′ (reverse primer). PCR was performed on mixtures with a final volume of 100 *μ*L containing 20 *μ*L of cytokines or *β*-actin cDNA using a DNA thermocycler (Eppendorf, Hamburg, Germany) under the following conditions: 35 cycles of 30-second denaturation at 94°C, 45-second annealing at 66°C for TNF-*α*, 63°C for IL-6, or 58°C for IL-1*β* and *β*-actin, and 1-minute extension at 72°C. The PCR products were visualized on a 1.2% agarose gel stained with ethidium bromide.

### 2.5. Nuclear protein preparation and electrophoretic mobility gel shift assay (EMSA)

THP-1 cells were cultured in a dish in 1% FBS for 24 hours and then stimulated with LP (3 *μ*g/mL) at indicated time intervals. The cells were collected on ice before isolation of nuclear extracts by the protocol reported by Muller and Homaidan [17, 18]. Briefly, the cells (10^6^/mL) were washed with ice-cold PBS, suspended in 200 *μ*L of lysis buffer (10 mM HEPES [pH7.9], 10 mM KCl, 0.1 mM EDTA, 0.1 mM EGTA, 1 mM DTT) and allowed to swell on ice for 15 minutes, and then 12.5 *μ*L of 10% Nonidet P-40 was added. The tube was then mixed thoroughly with a Vortex mixer for 10 seconds prior to centrifugation (20,000 g) at 4°C for 8 minutes. The nuclear pellets thus obtained were resuspended in 25 *μ*L of ice-cold nuclear extraction buffer (20 mM HEPES [pH7.9], 400 mM NaCl, 1 mM EDTA, 1 mM EGTA, 1 mM DTT) and kept on ice for 15 minutes with intermittent agitation. The samples were subjected to centrifugation for 5 minutes at 4°C, and the supernatant was stored at −70°C after measurement of its protein content with the Bio-Rad protein assay kit (Bio-Rad, Munich, Germany).

The detection of the activated NF-*κ*B in the nuclei of unstimulated and stimulated cells was completed by using a biotin-labeled EMSA kit (Viagene, Ningbo , China), according to the manufacture's instruction. The consensus NF-*κ*B oligonucleotides included in the kit was 5′ -AGT TGA GGG GAC TTT CCC AGG C-3′ .

### 2.6. Measurement of cell apoptosis

Two different techniques were used as follows. (1) AO-EB staining, an exclusion dye method which enables to differentiate between live, early-apoptotic, late-apoptotic, and necrotic cells [19]. At 12 hours after induction of apoptosis, cells were collected, centrifuged at 500 g for 10 minutes, and resuspended in 100 *μ*L PBS. Samples of 25 *μ*L from each culture were stained with AO-EB (final concentration 1 *μ*g/mL for each AO and EB), and observed under a fluorescence microscope. At least 200 cells were randomly counted in each sample (in duplicates), and the percentage of apoptotic cells was calculated; (2) Annexin-V-FITC-PI staining, which detects the exposure of phosphatidylserine (PS) to the external leaflet of the plasma membrane in early apoptosis, and enables to differentiate between live, early apoptotic, and dead cells (it does not differentiate between late apoptotic and necrotic cells) [20]. At 12 hours after induction of apoptosis, cells were washed with PBS, centrifuged and resuspended in 400 *μ*L of a binding bufter (10 mM HEPES/NaOH, 140 mM NaCl, 2.5 mM CaCl_2_, PH7.4). Annexin-V-FITC (5 *μ*L; 10 *μ*g/mL) was added to a sample of 195 *μ*L of cell suspension, mixed, incubated for 10 minutes at room temperature in the dark, washed with PBS, and resuspended in 190 *μ*L of binding buffer containing 10 *μ*L of PI (1 *μ*g/mL). The double-stained cells were analyzed by the FACS within 10 minutes (10^4^ cells/sample).

In all measurements of apoptosis, the percentage of cells that underwent apoptosis was determined by subtracting the percentage of spontaneous apoptosis (unstimulated cells) from the total apoptosis (stimulated cells).

### 2.7. Statistical analysis

Data obtained from the three independent experiments were expressed as mean±SE. The data were analyzed by a one-way ANOVA test followed by an independent-samples *t* test using SPSS software. A *P*-value of less than 0.05 was considered significant.

## 3. RESULTS

### 3.1. Marked production of cytokines in THP-1 by M. genitalium LP

**Figure 1:**
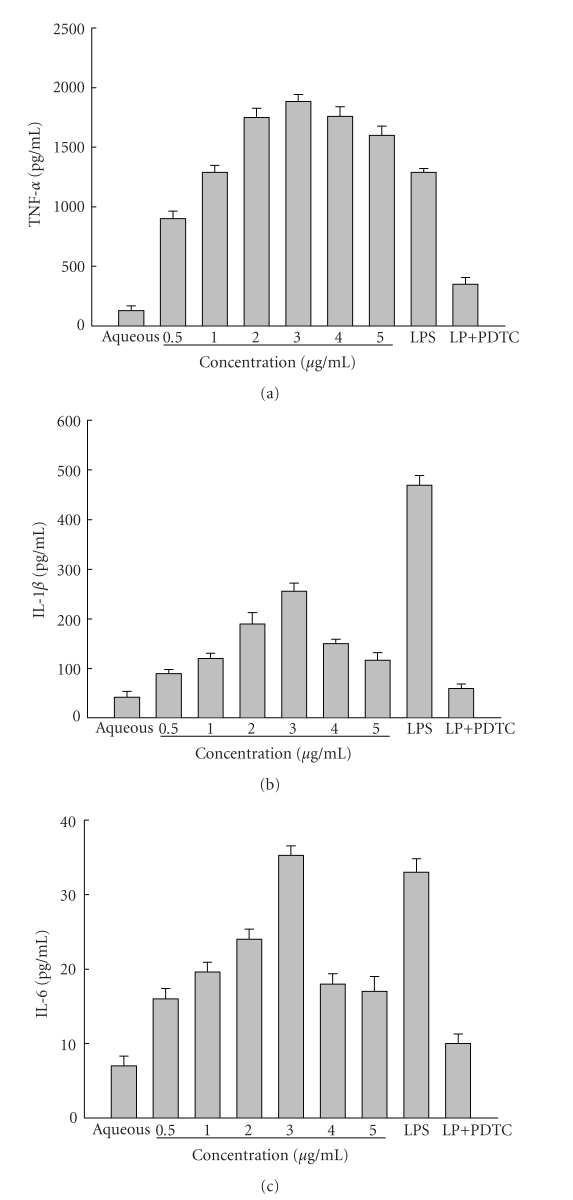
The effect of different concentrations of LP on the production of TNF-*α* (a), IL-6 (b), and IL-1*β* (c) in THP-1 cells. Dose-dependent induction of TNF-*α* (a), IL-6 (b), and IL-1*β* (c) was seen in THP-1 cells after 24 hours of stimulation with 0.5 to 5 *μ*g/mL of LP, 3 *μ*g/mL of LP in combination with 25 *μ*M PDTC, or 0.1 *μ*g/mL LPS. In this experiment THP-1 cells stimulated with aqueous phase were used as the negative control. Proinflammatory cytokine levels were determined by ELISA as indicated in Section 2. The results were representative of three independent experiments.

**Figure 2:**
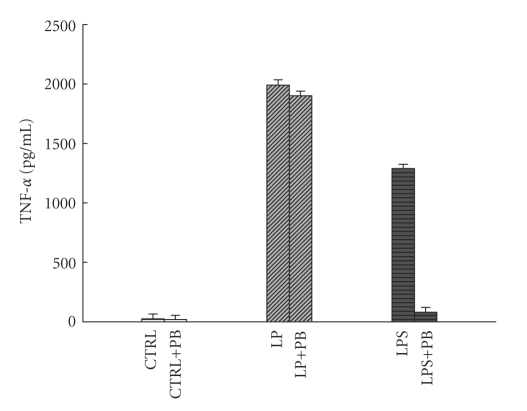
Effects of polymyxin B on LP or LPS-induced TNF-*α* production in THP-1 cells. LP or LPS was pretreated by 100 *μ*g/mL polymyxin B (PB) for 2 hours before challenging THP-1 cells. LP (3 *μ*g/mL) or LPS (100 ng/mL) was added to the medium. After being incubated for 24 hours, cells were harvested, and the concentration of TNF-*α* in the medium was determined by ELISA as described in Section 2.

LP prepared from *M. genitalium* stimulated THP-1 cells to produce TNF-*α*, IL-1*β*, and IL-6 in dose-dependent manner (Figure 1). The dose-response bar revealed a 3 *μ*g/mL optimal concentration of LP for the induction of TNF-*α* [(1885.29 ± 58.62) pg/mL], IL-1*β* [(256.20 ± 16.030) pg/mL], and IL-6 [(35.29 ± 1.26) pg/mL], leading to cytokine concentration similar to those obtained with 0.1 *μ*g/mL LPS [TNF-*α* (1288.96 ± 34.34) pg/mL, IL-1*β* (469.36 ± 21.11) pg/mL, IL-6 (33.01 ± 1.81) pg/mL, *P* > .05]. Interestingly, when the concentration of LP was increased from 3 *μ*g/mL to 5 *μ*g/mL, the level of cytokines decreased. The contamination of LP by LPS was not responsible for this since polymyxin B pretreated LP had no effect on TNF-*α* production (Figure 2).

### 3.2. Induction of TNF-*α*, IL-1*β*, and IL-6 mRNA expression after treatment with LP

**Figure 3:**
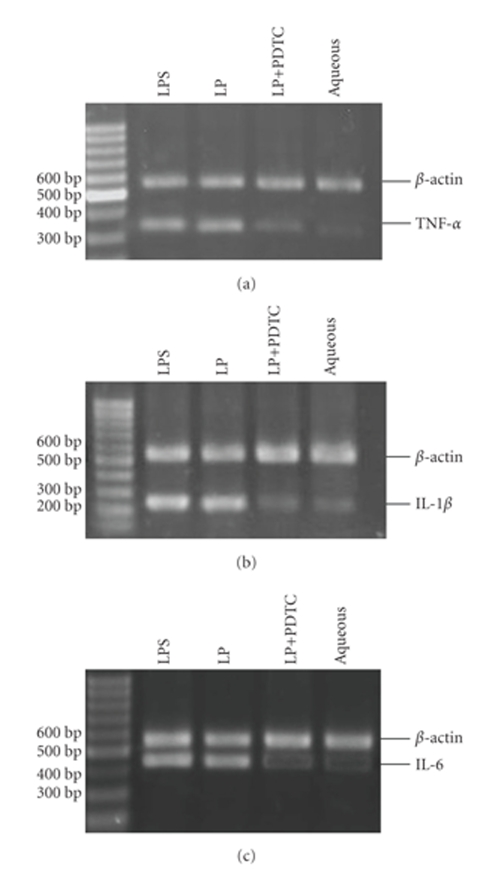
The effect of different groups on the expression of TNF-*α* (a), IL-6 (b), and IL-1*β* (c) mRNA. THP-1 cells were stimulated for 18 hours and the mRNA levels were determined by RT-PCR. The levels of mRNA expression were inhibited upon the introduction of 25 *μ*M PDTC. The bottom photograph depicts the result of RT-PCR for mRNA, and the top photograph depicts the *β*-actin positive control. Lane M: 100 bp DNA marker; lane 1: 0.1 *μ*g/mL LPS; lane 2: 3 *μ*g/mL of LP; lane 3: 3 *μ*g/mL of LP in combination with 25 *μ*M PDTC; lane 4: 100 *μ*L aqueous phase (negative control).

Because the cytokine mRNA are regulated mainly at the transcriptional level, we examined the expression levels of TNF-*α*, IL-1*β*, and IL-6 mRNA using RT-PCR. As shown in Figure 3, the addition of 3 *μ*g/mL LP after 18-hour stimulation increased these three genes' mRNA expression and *β*-actin as an internal control was similar in all samples.

### 3.3. PDTC-induced downregulation of TNF-*α*, IL-1*β*, and IL-6 production and its mRNA expression

We found that the NF-*κ*B inhibitor PDTC significantly inhibited the production of TNF-*α*, IL-1*β*, and IL-6 in THP-1 cells stimulated by *M. genitalium* LP. As shown in Figure 1, the levels of TNF-*α*, IL-1*β*, and IL-6 in the conditioned media from cells treated with 3 *μ*g/mL of LP in combination with 25 *μ*M PDTC were significantly lower than in media from cells treated 3 *μ*g/mL of LP alone (*P* < .05). The effects of PDTC on the expression of TNF-*α*, IL-1*β*, and IL-6 mRNA showed similar patterns in LP-stimulated cells, as determined by RT-PCR (Figure 3).

### 3.4. M. genitalium LP induced NF-*κ*B activation by EMSA

**Figure 4:**
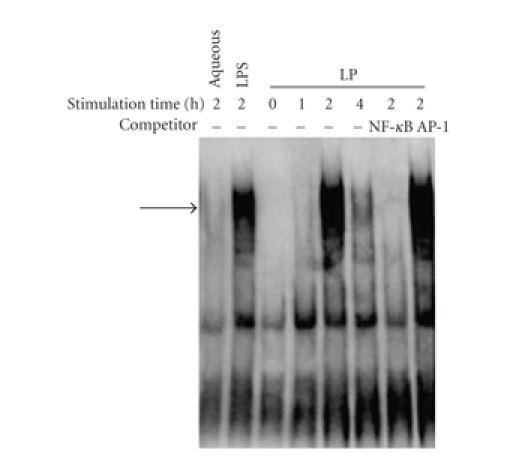
The activity of NF-*κ*B in different groups was examined by EMSA. NF-*κ*B activation was measured by EMSA using a biotin-labeled oligonucleotide encompassing the NF-*κ*B consensus motif. THP-1 cells were stimulated with *M. genitalium* LP (3 *μ*g/mL) at different time intervals (0, 1, 2, and 4 hour(s)). THP-1 cells treated with 100 *μ*L aqueous phase and 0.1 *μ*g/mL LPS were used as control. The specificity of DNA binding was assessed by preincubating extracts with unlabeled specific (NF-*κ*B) or unspecific (AP-1) competitor oligonucleotide. The arrow indicates specific NF-*κ*B band.

In this study, we investigated whether *M. genitalium* LP was capable of triggering NF-*κ*B activation. For this purpose, the THP-1 cells were stimulated with LP at different time intervals, NF-*κ*B DNA binding activities in nuclear extracts were assessed by nonradioactive EMSA as described in Section 2. As depicted in Figure 4, *M. genitalium* LP could activate NF-*κ*B in THP-1 cells: the DNA binding activities were maximal by 2 hours of stimulation and then declined. The specificity of NF-*κ*B DNA binding was verified by competition analysis with an excess of unlabeled specific or unspecific oligonucleotides.

### 3.5. M. genitalium LP induced cell apoptosis by Annexin-V-FITC-PI and AO-EB staining

**Figure 5:**
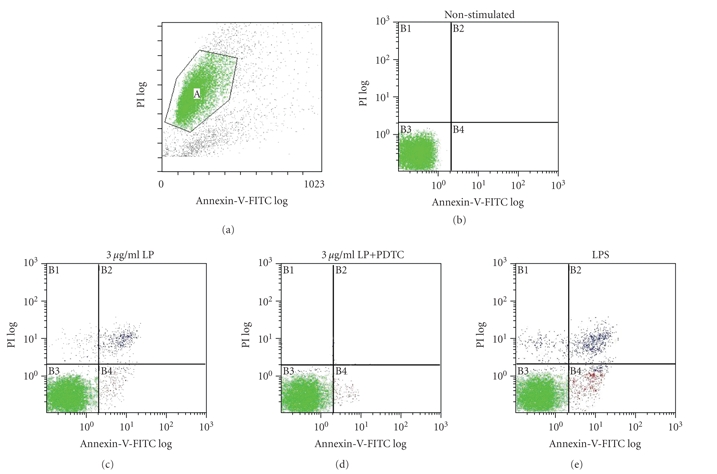
Cell apoptosis of different groups was detected by Annexin-V-propidium iodide staining. THP-1 cells were stimulated with 3 *μ*g/mL of LP, 3 *μ*g/mL of LP in combination with 25 *μ*M PDTC, or 0.1 *μ*g/mL LPS for 12 hours, stained with Annexin-V-FITC-PI and analyzed by FACS. Double negative staining represents living cells (c), positive staining for Annexin-V-FITC, and negative staining for PI represent the early apoptotic stage (e), and double-positive staining represents the late apoptotic stage (b).

The percentage of apoptotic cells was examined by two techniques as follows. (1) a typical experiment of Annexin-V-FITC-PI staining was shown in Figure 5. By this technique, necrotic and late apoptosis cells could not be distinguished from each other, so we concluded that the double-positive cells (for Annexin-V-FITC and PI) represented the late apoptotic cells and not necrotic ones. The results obtained in the three independent experiments showed approximately 14.23 ± 1.56% reduction in the percentage of the apoptotic cells induced by *M. genitalium* LP, in comparison with LPS control (16.53 ± 1.68%) and LP in combination with 25 *μ*M PDTC-induced cells (2.79 ± 0.46%). (2) AO-EB staining, which distinguishes between apoptotic cells by the morphological changes in the nucleus (lack of DNA condensation in necrotic cells as supposed to apoptotic cells), was used to determine whether *M. genitalium* LP induced apoptosis or necrosis. The apoptosis was reduced by about 61% in THP-1 cells infected with *M. genitalium* LP in combination with 25 *μ*M PDTC (15.34 ± 3.94%), in comparison with *M. genitalium* LP-infected cells (38.50 ± 4.46%) and LPS-treated cells (39.60 ± 4.45%) (Table 1).

## 4. DISCUSSION

Although the molecular basis of mycoplasma pathogenicity remains unclear, modulatory effect on the immune system induced by LP appears to play an important role in the development of mycoplasma-associated diseases [21]. One of the best documented effects of mycoplasma is the induction of numerous cytokines by monocytic cells [9]. For example, *M. fermentans*-derived LP has been demonstrated to induce inflammatory mediators such as TNF-*α*, IL-1*β*, and IL-6 released from mouse macrophages [4, 22]. In the present study, we have demonstrated that lipoproteins derived from the human *M. genitalium* are capable of inducing THP-1 cells to produce proinflammatory cytokines and to induce apoptosis by activating NF-*κ*B.

In an attempt to clarify the potential pathogenicity of *M. genitalium*, we have demonstrated in this study that LP from *M. genitalium* could trigger THP-1 cells to produce TNF-*α*, IL-1*β*, and IL-6 in a dose-dependent manner. However, when the concentration of LP increased from 3 to 5 *μ*g/mL, the level of inflammatory cytokines decreased. This may be explained by assuming that excessively high concentrations of LP may be toxic to THP-1 cells, and thus, decreased inflammatory cytokine production [8]. We have also found that PDTC, an inhibitor of NF-*κ*B [23], could significantly inhibit THP-1 cells treated with LP from producing inflammatory cytokines, and inhibit the expression of their mRNA. TNF-*α*, IL-6, and IL-1*β* are important inflammatory mediators: they can stimulate rapid neutrophil influx, and these cells are effective mediators of host defense until antigen-specific mechanisms are induced to eliminate the pathogen. However, the stimulation of cytokine production could also influence the development of inflammatory reactions inevitably, which may directly or indirectly contribute to disease pathogenesis and tissue damage [24].

Recent studies have demonstrated that there are NF-*κ*B binding sites in the 5′ transcriptional regulation regions of the cytokine gene. NF-*κ*B is known as a widespread rapid-response transcription factor that is normally expressed in the cytoplasm of a variety of cells [25, 26]. Since the induction of gene-specific recognition elements located in the upstream promoter region, we investigated whether the production of TNF-*α*, IL-1*β*, and IL-6 and the expression of their mRNA in THP-1 cells treated with *M. genitalium* LP were associated with the activation of NF-*κ*B. By using electrophoretic mobility shift and transactivation assays, we have clearly demonstrated that LP can induce the transcriptional activation of NF-*κ*B; the activation peaked 2 hours after stimulation. The above results indicate that LP from *M. genitalium* are potent activators of NF-*κ*B, and NF-*κ*B activation may be of great importance for inducing the production of TNF-*α*, IL-1*β*, and IL-6 and the expression of their mRNA.

Apoptosis is a major form of cell death, characterized initially by a series of stereotypical morphological changes. These changes reflect complex biochemical events carried out by a family of cysteine proteases called caspases [27–29]; but a question remains to be answered whether the *M. genitalium* LP could induce apoptosis by macrophages in addition to inducing inflammatory cytokine production. In this study, cell apoptosis was detected in THP-1 cells treated with the *M. genitalium* LP by Annexin V-FITC-PI and AO-EB staining, and the apoptosis-inducing activity was inhibited by PDTC, which demonstrated that *M. genitalium* LP-induced THP-1 cell death was partially associated with the activation of NF-*κ*B [4]. In terms of infectious diseases, apoptosis appears to be one of the defense mechanisms against microbes hiding in cells which do not necessarily possess any mechanism against them. However, excessive immune cells apoptosis may affect the immune response in the primary infection site, and thus make it easy for mycoplasma to diffuse [30, 31]. It is very likely that apoptosis-related cytokines, such as TNF-*α* produced by macrophages and lymphocytes in response to mycoplasmal lipoproteins, play an important role in the expression of the cytotoxicity. This speculation is supported by findings that *M. fermentans* enhances concanavalin A-induced apoptosis of mouse splenic T-cells and that TNF-*α* plays a key role in the activity [32]. However, whether these cytokines exert some effects on the cytotoxicity of *M. genitalium* LP to THP-1 cells is still unknown. Further studies are in progress to clarify the molecular mechanism.

The above results indicate that LP from *M. genitalium* are potent activators of NF-*κ*B, and that NF-*κ*B activation may be of great importance for inducing the production of proinflammatory cytokines and its mRNA and apoptosis following stimulation with LP from *M. genitalium*. Since proinflammatory cytokines play important roles in the pathogenesis of infectious disease sequelae, the success of anticytokine therapy in the inflammatory reaction may relieve the abnormal immune response brought about by mycoplasma infection. It is now clear that NF-*κ*B were involved in this modulation, and these findings contribute to unraveling the complex mechanisms of immune reactivity to mycoplasma infection and may ultimately prove useful in the development of new therapeutic strategies to prevent tissue damage in mycoplasma-associated diseases [33].

## Figures and Tables

**Table 1:** Cell apoptosis induced by different groups, as determined by fluorescence microscopy. Values, quantified by random counting of acridine orange-ethidium bromide-stained cells, were mean ± standard deviation of data from the three independent experiments. Percentages represent the different stages of cell death. (*P* < .05, as determined by ANOVA single factor).

Treatment	% of live cells	% of necrotic	% of apoptotic cells		
			Early	Late	Total
aqueous	88.13 ± 4.71	2.53 ± 1.12	7.01 ± 3.15	2.33 ± 1.42	9.34 ± 4.32
LP	52.68 ± 5.24	8.82 ± 2.49	14.42 ± 3.81	24.08 ± 6.25	38.50 ± 4.46^a^
LP+PDTC	80.56 ± 2.73	4.10 ± 1.56	9.85 ± 4.53	5.49 ± 2.78	15.34 ± 3.94^b^
LPS	49.05 ± 3.14	11.35 ± 3.96	13.37 ± 4.87	26.23 ± 5.72	39.60 ± 4.45^c^

LP versus aqueous treatment: ^a^ *P* < .05; LP+PDTC versus LP treatment: ^b^ *P* < .05; LPS versus aqueous treatment: ^c^ *P* < .05.
